# Genomic characteristics of clinical multidrug-resistant *Proteus* isolates from a tertiary care hospital in southwest China

**DOI:** 10.3389/fmicb.2022.977356

**Published:** 2022-08-24

**Authors:** Ying Li, Qian Liu, Yichuan Qiu, Chengju Fang, Yungang Zhou, Junping She, Huan Chen, Xiaoyi Dai, Luhua Zhang

**Affiliations:** ^1^The School of Basic Medical Science and Public Center of Experimental Technology, Southwest Medical University, Luzhou, Sichuan, China; ^2^Immune Mechanism and Therapy of Major Diseases of Luzhou Key Laboratory, School of Basic Medical Science, Southwest Medical University, Luzhou, Sichuan, China; ^3^Department of Clinical Laboratory, The Affiliated Traditional Chinese Medicine Hospital, Southwest Medical University, Luzhou, Sichuan, China

**Keywords:** *Proteus*, SXT/R391 ICEs, *bla*_NDM-1_, *bla*_KPC-2_, plasmid

## Abstract

Multidrug-resistant (MDR) *Proteus*, especially those strains producing extended-spectrum β-lactamases (ESBL) and carbapenemases, represents a major public health concern. In the present work, we characterized 27 MDR *Proteus* clinical isolates, including 23 *Proteus mirabilis*, three *Proteus terrae*, and one *Proteus faecis*, by whole-genome analysis. Among the 27 isolates analyzed, SXT/R391 ICEs were detected in 14 strains, and the complete sequences of nine ICEs were obtained. These ICEs share a common backbone structure but also have different gene contents in hotspots and variable regions. Among them, ICE*Pmi*Chn2826, ICE*Pmi*Chn2833, ICE*Pmi*Chn3105, and ICE*Pmi*Chn3725 contain abundant antibiotic resistance genes, including the ESBL gene *bla*_CTX-M-65_. The core gene phylogenetic analysis of ICEs showed their genetic diversity, and revealed the cryptic dissemination of them in *Proteus* strains from food animals and humans on a China-wide scale. One of the isolates, FZP3105, acquired an NDM-1-producing MDR plasmid, designated pNDM_FZP3105, which is a self-transmissible type 1/2 hybrid IncC plasmid. Analysis of the genetic organization showed that pNDM_FZP3105 has two novel antibiotic resistance islands bearing abundant antibiotic resistance genes, among which *bla*_NDM-1_ is located in a 9.0 kb ΔTn*125* bracketed by two copies of IS*26* in the same direction. In isolates FZP2936 and FZP3115, *bla*_KPC-2_ was detected on an IncN plasmid, which is identical to the previously reported pT211 in Zhejiang province of China. Besides, a MDR genomic island PmGRI1, a variant of PmGRI1-YN9 from chicken in China, was identified on their chromosome. In conclusion, this study demonstrates abundant genetic diversity of mobile genetic elements carrying antibiotic resistance genes, especially ESBL and carbapenemase genes, in clinical *Proteus* isolates, and highlights that the continuous monitoring on their transmission and further evolution is needed.

## Introduction

*Proteus* spp., belonging to the family of Morganellaceae of the order Enterobacterales (Adeolu et al., 2016), are widespread in the environment, and also inhabit in the intestines of humans and animals (O’Hara et al., 2000). Currently, ten species were included in the genus *Proteus*, among which, *Proteus mirabilis* is the most frequently isolated from clinical samples, and represents a major cause of urinary tract infections (Schaffer and Pearson, 2015). *Proteus* are intrinsically resistant to polymyxins, nitrofurans, tigecycline, and tetracycline, and are naturally susceptible to aminoglycosides, fluoroquinolones, and trimethoprim-sulfamethoxazole (Korytny et al., 2016). Multidrug-resistant (MDR), to 3 or more classes of antimicrobial agents (Falagas and Karageorgopoulos, 2008) *Proteus* have been increasingly reported, which generally produce extended-spectrum β-lactamases (ESBLs) and AmpC-type cephalosporinase, and show co-resistance to fluoroquinolones, aminoglycosides, and sulfamethoxazole-trimethoprim (Tumbarello et al., 2012; Korytny et al., 2016). Carbapenems are one of the last-resort antibiotics to treat severe infections caused by MDR *Proteus*, while the identification of carbapenemases, such as KPC-2 (Hu et al., 2012), NDM-1 (He et al., 2021), OXA-48 (Fursova et al., 2015), OXA-23, and OXA-58 (Bonnin et al., 2020) in *Proteus* causes great clinical concern. Plasmid-mediated horizontal gene transfer plays an important role in the dissemination of carbapenemase genes in *Proteus* (Hua et al., 2020; Li et al., 2021).

Resistance genes on the chromosome are usually clustered into structures named genomic islands (GIs), which are distinct regions of a bacterial chromosome that have been acquired *via* horizontal transfer (Partridge et al., 2018). Integrating and conjugative elements (ICEs) are a specific GI with mobility functions that can be integrated into the host chromosome, and also excised as a circular intermediate to be self-transferred to a recipient cell *via* conjugation (Johnson and Grossman, 2015). The large SXT/R391 family ICEs are characterized by a conserved integrase, which catalyzes their integration into the 5′ end of the chromosomal *prfC* gene (encoding the peptide chain release factor 3) by site-specific recombination (Bioteau et al., 2018). SXT/R391 ICEs share 52 nearly identical core genes that mediate integration/excision, conjugative transfer, and regulation, and also contain variable DNA regions, dubbed hotspots (HS) 1–5 and variable regions (VR) I-IV (Wozniak et al., 2009), wherein SXT/R391 acquire new variable DNA sequences that confer element-specific phenotypes, such as resistance to antibiotics (Rodriguez-Blanco et al., 2012). Several SXT/R391 elements have been identified in *Proteus*, and they serve as vehicles of clinical important resistance genes, such as *bla*_CMY-2_ (Harada et al., 2010), *bla*_NDM-1_ (He et al., 2021), *tet*(X6) (Peng et al., 2020) and *bla*_CTX-M-65_ (Lei et al., 2018), which constitutes a severe concern. At present, SXT/R391 ICEs have been widely studied in food-producing animals in China (Lei et al., 2016, 2018; Wang et al., 2021), while their prevalence and genetic characteristics in the clinical isolates are not well understood.

The present study was conducted to characterize MDR *Proteus* clinical isolates from a tertiary care hospital in Sichuan province, China. We performed whole-genome sequencing and analysis to investigate their genetic features about resistance determinants, with a focus on the diversity of genetic structure of SXT/R391 ICEs and the genetic contexts of carbapenemase-encoding genes. Also, the transfer capability of ICEs and carbapenemase-encoding plasmids were determined by conjugation assays.

## Materials and methods

### Bacterial isolates

*Proteus* isolates were isolated from clinical specimens of patients at the Affiliated Traditional Chinese Medicine Hospital of Southwest Medical University, in Sichuan Province, China, from January to December of 2021. This study was approved by the Ethics Committee of the Affiliated Traditional Chinese Medicine Hospital of Southwest Medical University. Written informed consent from the patients was exempted for this study, since the present study only focused on bacteria and the strains were isolated as a part of the routine hospital laboratory procedures. Species identification was carried out by 16S rRNA gene sequencing analysis (Lane, 1991). Antibiotic susceptibility testing was performed using the Biomerieux Vitek-2 system, and the results were interpreted by the clinical breakpoints defined by the Clinical and Laboratory Standards Institute standards for Enterobacterales (CLSI, M100). *Escherichia coli* strain ATCC 25922 was used as the quality control. The antibiotics used in this study was provided in Supplementary Table S1. The minimum inhibitory concentrations (MICs) of meropenem against the isolates were determined using the microdilution broth methods and the disk diffusion methods of Bauer and Kirby following recommendations of the CLSI. MDR strains were defined as non-susceptibility to at least one agent in three or more of the following antibiotic groups: β-lactam-β-lactam inhibitor combinations, cephalosporins, fluoroquinolones, aminoglycosides or sulfamethoxazole and trimethoprim (Korytny et al., 2016).

### Genomic DNA sequencing and data analysis

Genomic DNA was prepared using the QIAamp DNA Mini Kit (Qiagen) following the manufacturer’s guidelines. Whole genome sequencing was performed on the HiSeq 2000 (Illumina, San Diego, CA, United States) platform using a paired-end library with an insert size of 150 bp by the Beijing Tsingke Bioinformatics Technology Co. Ltd. Four isolates (FZP2936, FZP3115, FZP3105 and FZP2826) were additionally sequenced on the long-read MinION sequencer (Nanopore, Oxford, United Kingdom). Both the long MinION reads and short Illumina reads were *de novo*-assembled using Unicycler under the conservative mode (Wick et al., 2017). Pilon was employed to correct the assembled contigs with Illumina reads (Walker et al., 2014). Annotation was carried out using the RAST tools (Aziz et al., 2008) combined with BLASTp/BLASTn searches against the UniProtKB/SwissProt database (Boutet et al., 2016).

The identification of *Proteus* species was performed by average nucleotide identity (ANI) analysis with JSpeciesWS.^1^ Plasmid incompatibility types and multilocus sequence typing were identified using PlasmidFinder 2.1 (95%, minimum threshold for identity; 60%, minimum coverage) and pMLST 2.0 (Carattoli and Hasman, 2020). Antibiotic resistance genes (ARGs), insertion elements (ISs) and integrons were predicted using ResFinder (90%, minimum threshold for identity; 60%, minimum coverage; Bortolaia et al., 2020), ISfinder (Siguier et al., 2006) and INTEGRALL (Moura et al., 2009). The presence of SXT/R391 ICE was screened by targeting the conserved integrase gene (*int*_SXT_) from the whole sequenced genomes. The contigs of SXT/R391 ICE were extracted and assembled against the reference ICE in the genome of FZP3105, with gaps between contigs closed by PCR and Sanger sequencing. Plasmids/ICEs similar to those in this study were identified by a BLASTn search in the GenBank database using whole plasmid/ICE sequences. Linear sequence alignment was performed using BLAST and visualized with Easyfig 2.2.3 (Sullivan et al., 2011).

### Phylogenetic analysis

The genome sequences of other representative *Proteus* isolates in China from the literature were retrieved from the GenBank. Genetic relationship between the *Proteus* isolates in this study and these reference strains was assessed based on single nucleotide polymorphisms (SNPs) in their core genomes, as previously described with minor modification (Li et al., 2022). Briefly, Genomes were annotated using Prokka, and the generated GFF3 files were piped into Roary to create a core genome alignment. SNPs were extracted using snp-sites v2.3.2. A maximum-likelihood phylogenetic tree based on the SNPs was constructed using FastTree version 2.1.10 under the GTR model. Similarly, phylogenetic analysis of the ICEs in this study and other representative ICEs from the literature was carried out based on SNPs in their conserved regions. The presence of ARGs in the bacterial genomes was determined by ResFinder, and detailed information of isolates was annotated on the trees using iTOL.^2^

### Transferability assay

Conjugation experiments were performed using broth-based method with the rifampin-resistant *E. coli* strain EC600, and azide-resistant *E. coli* strain J53 as the recipients, as described previously with minor modification (Li et al., 2021). After the donor strain and recipient were grown to exponential stage (the optical density at 600 nm reaches ~0.5), mix them at a ratio of 1:1, and incubate at 37°C for 24 h. Transconjugants were selected on Luria-Bertani (LB) agar plates containing 4 μg/ml meropenem, 16 μg/ml gentamicin or 2 μg/ml tigecycline plus 400 μg/ml rifampin or 150 μg/ml sodium azide. The presence of *bla*_NDM-1_, or SXT/R391 ICE was confirmed by PCR using the primers *bla*_NDM_-F 5′ -ATTTACTAGGCCTCGCATTTGC-3′ /*bla*_NDM_-R 5′-GCCTCTGTCACATCGAAATCG-3′, and sxtintF 5′-TCGATGATGGTCTCTAGCTG-3′ /5′-TCAGTTAGCTGGCTCGATGC-3′ (Sato et al., 2020), respectively, with the following conditions: 95°C for 5 min, and 30 cycles of amplification consisting of 30 s at 95°C, 30 s at 53°C, and 1 min 30 s at 72°C, followed by a final elongation step for 5 min at 72°C.

## Results and discussion

### Sources, resistance phenotype, and genotype of MDR *Proteus* isolates

54 *Proteus* strains were isolated from clinical specimens, and 27 of them were identified as MDR strains (Table 1). These MDR isolates were obtained from urine (*n* = 13, 48.1%), wound secretion (*n* = 8, 29.6%), sputum (*n* = 2, 7.4%), blood (*n* = 2, 7.4%), sanies (*n* = 1, 3.7%), and drainage (*n* = 1, 3.7%). ANI analysis indicated that 27 MDR *Proteus* strains belong to *P. mirabilis* (*n* = 23), *Proteus terrae* (*n* = 3), and *Proteus faecis* (*n* = 1). In addition to their intrinsic resistance profiles, these MDR *Proteus* isolates also showed high levels of resistance to ampicillin (*n* = 27, 100%), cefaloti (*n* = 19, 70.4%), ceftriaxone (*n* = 17, 63.0%), cefotaxime (*n* = 19, 70.4%), ampicillin/sulbactam (*n* = 18, 66.6%), sulfamethoxazole-trimethoprim (*n* = 26, 96.3%), moxifloxacin (*n* = 18, 66.6%), ciprofloxacin (*n* = 21, 77.7%), levofloxacin (*n* = 18, 66.6%), gentamicin (*n* = 13, 48.1%), and tobramycin (*n* = 11, 40.7%). Some MDR isolates were also resistant to aztreonam (*n* = 6, 22.2%), norfloxacin (*n* = 9, 33.3%), imipenem (*n* = 5, 18.5%), meropenem (*n* = 3, 11.1%), ceftazidime (*n* = 2, 7.4%), and piperacillin/tazobactam (*n* = 2, 7.4%; Table 1). Notably, all strains remained susceptible to amikacin.

**Table 1:** Microbiological and molecular characteristics of 27 MDR *Proteus* isolates.

Strain	Species	Specimen	Gender/age (year)	ICE/variants of PmGRI1	Resistance phenotype
FZP1665	*Proteus mirabilis*	Urine	F/49	PmGRI1	AMP, CIP, GEN, NIT, SXT, TCY, TGC
FZP2056	*Proteus mirabilis*	Urine	M/75	ICE	AMP, CIP, CZO, LVX, MFX, NIT, NOR, SAM, SXT, TCY, TGC
FZP2095	*Proteus terrae*	Wound secretion	M/56		AMP, ATM, CAZ, CIP, CRO, CTX, CXM, CZO, FEP, FOX, SAM, SXT, TGC
FZP2128	*Proteus mirabilis*	Wound secretion	M/41	PmGRI1	AMP, GEN, SAM, SXT, TOB
FZP2958	*Proteus mirabilis*	Urine	M/74	ICE	AMP, CEP, CIP, CPD, CRO, CTX, CXA, CXM, CZO, LVX, MFX, NIT, SAM, SXT, TCY, TGC
FZP3803	*Proteus mirabilis*	Urine	M/35	ICE	AMP, CEP, CIP, CPD, CRO, CTX, CXA, CXM, CZO, GEN, LVX, MFX, NIT, PIP, SAM, SXT, TCY, TGC, TOB
FZP4264	*Proteus mirabilis*	Urine	M/70	PmGRI1	AMC, AMP, ATM, CEP, CIP, CPD, CRO, CTX, CXA, CXM, CZO, CZX, FEP, GEN, LVX, MFX, NIT, NOR, PIP, SAM, SXT, TCY, TGC, TOB
FZP4280	*Proteus mirabilis*	Urine	M/80	ICE, PmGRI1	AMP, CEP, CIP, CPD, CRO, CTX, CXA, CXM, CZO, LVX, MFX, NIT, PIP, SXT, TCY, TGC
FZP4349	*Proteus terrae*	Wound secretion	F/58	ICE	AMP, CEP, CXA, CXM, CZO, NIT, SXT, TCY, TGC
FZP4423	*Proteus mirabilis*	Blood	M/82	ICE	AMC, AMP, CEP, CIP, CPD, CTX, CXA, CXM, CZO, CZX, GEN, IPM, LVX, MFX, NOR, PIP, SAM, SXT, TCY, TGC, TOB
FZP4515	*Proteus mirabilis*	Wound secretion	M/49	PmGRI1	AMP, CEP, CIP, CPD, CRO, CTX, CXA, CXM, CZO, GEN, LVX, MFX, NOR, PIP, SXT, TCY
FZP1097	*Proteus faecis*	Wound secretion	M/76		AMP, ATM, CIP, CRO, CTX, SXT
FZP1177	*Proteus mirabilis*	Sputum	F/67	PmGRI1	AMP, ATM, CIP, CRO, CTX, CXM, CZO, FEP, GEN, MFX, NOR, LVX, SXT, TOB
FZP1611	*Proteus mirabilis*	Sputum	M/66		AMP, ATM, CEP, CIP, CPD, CRO, CTX, CXA, CXM, CZO, CZX, FEP, GEN, LVX, MFX, NIT, PIP, SAM, SXT, TCY, TGC, TOB
FZP2024	*Proteus mirabilis*	Urine	M/77	ICE, PmGRI1	AMP, CIP, GEN, LVX, MFX, NIT, SXT, TCY, TGC
FZP2561	*Proteus mirabilis*	Urine	F/49	ICE	AMP, CEP, CPD, CRO, CTX, CXA, CXM, CZO, NIT, PIP, SXT, TCY
FZP2833	*Proteus mirabilis*	Drainage	F/49	ICE	AMP, CEP, CIP, CPD, CRO, CTX, CXA, CXM, CZO, LVX, MFX, PIP, SAM, SXT, TCY, TGC
FZP2937	*Proteus mirabilis*	Wound secretion	M/32	ICE, PmGRI1	AMC, AMP, CEP, CIP, CPD, CRO, CTX, CXA, CXM, CZO, LVX, MFX, NOR, PIP, SAM, SXT, TCY, TGC
FZP3043	*Proteus mirabilis*	Urine	F/37	ICE	AMC, AMP, ATM, CEP, CPD, CTX, CXA, CXM, CZO, CZX, NIT, SAM, SXT, TCY, TGC, TOB
FZP3105	*Proteus mirabilis*	Sanies	M/32	ICE, PmGRI1	AMC, AMP, CAZ, CEP, CIP, CPD, CRO, CTX, CXA, CXM, CZO, IPM, LVX, MFX, MEM, NOR, PIP, SAM, SXT, TCY, TGC
FZP3320	*Proteus mirabilis*	Urine	F/56		AMP, CIP, GEN, LVX, MFX, NIT, SAM, SXT, TCY, TGC
FZP3364	*Proteus terrae*	Wound secretion	F/82		AMP, CEP, CXA, CXM, CZO, TCY, TGC
FZP3406	*Proteus mirabilis*	Urine	F/52		AMC, AMP, CEP, CPD, CXA, CXM, CZO, GEN, NIT, SAM, SXT, TCY, TOB
FZP3725	*Proteus mirabilis*	Blood	M/81	ICE, PmGRI1	AMP, CEP, CIP, CPD, CRO, CTX, CXA, CXM, CZO, LVX, MFX, PIP, SAM, SXT, TCY, TGC
FZP2826	*Proteus mirabilis*	Urine	M/58	ICE	AMC, AMP, CEP, CIP, CPD, CRO, CTX, CXA, CXM, CZO, FEP, IPM, LVX, MFX, NIT, PIP, SAM, SXT, TCY, TGC, TOB
FZP2936	*Proteus mirabilis*	Urine	M/58	PmGRI1	AMC, AMP, CEP, CIP, CPD, CRO, CTT, CTX, CXA, CXM, CZO, ETP, GEN, IPM, LVX, MEM, MFX, NIT, NOR, PIP, SAM, SXT, TCY, TGC, TOB, TZP
FZP3115	*Proteus mirabilis*	Wound secretion	M/58	PmGRI1	AMC, AMP, CEP, CIP, CPD, CRO, CSL, CTT, CTX, CXA, CXM, CZO, ETP, GEN, IPM, LVX, MEM, MFX, NIT, NOR, PIP, SAM, SXT, TCY, TGC, TOB, TZP

AMC, amoxicillin/clavulanic acid; AMP, ampicillin; ATM, aztreonam; CIP, ciprofloxacin; CAZ, ceftazidime; CEP, cefalotin; CPD, cefpodoxime; CRO, ceftriaxone; CSL, cefoperazone/sulbactam; CTT, cefotetan; CTX, cefotaxime; CXA, cefuroxime axetil; CXM, cefuroxime; CZO, cefazolin; CZX, ceftizoxime; ETP, ertapenem; FEP, cefepime; FOX, cefoxitin; GEN, gentamicin; IPM, imipenem; LVX, levofloxacin; MEM, meropenem; MFX, moxifloxacin; NIT, nitrofurantoin; NOR, norfloxacin; PIP, piperacillin; SAM, ampicillin/sulbactam; SXT, trimethoprim/sulfamethoxazole; TCY, tetracycline; TGC, tigecycline; TOB, tobramycin; TZP, piperacillin/tazobactam.

**Figure 1:**
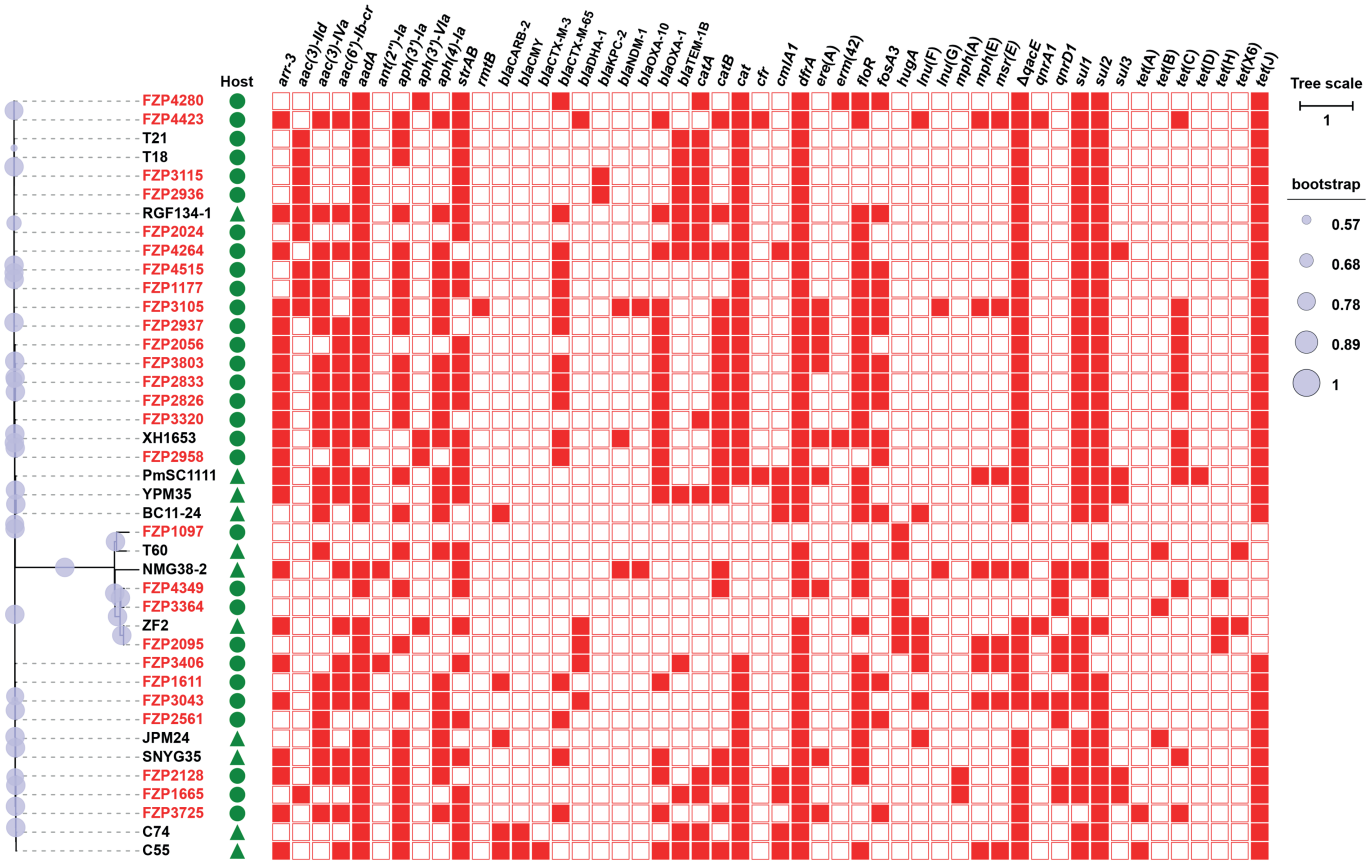
Phylogenetic analysis and antibiotic resistance gene (ARG) distribution of *Proteus* strains. Strains identified in this study are highlighted in red. The host of the isolate is indicated by triangles (animals) or circles (humans). The presence or absence of ARGs is indicated by filled or empty squares, respectively. The tree scale indicates substitutions per site. Detailed information of these strains is presented in Supplementary Table S4.

We sequenced the genomes of all 27 MDR isolates on the Illumina platform (Supplementary Table S2). Genomic analysis revealed that 57 different ARGs were detected in the 27 MDR *Proteus* strains, and 23 (85.2%) of them carried at least 13 ARGs (Figure 1). Of the detected β-lactamase genes, *bla*_CTX-M-65_, the only ESBL-producing gene, was the most prevalent (*n* = 13, 48.1%), followed by the non-ESBL *bla*_OXA-1_ (*n* = 12). Carbapenemase gene *bla*_KPC-2_ was found in two isolates, FZP2936 and FZP3115, in combination with *bla*_TEM-1B_. *bla*_NDM-1_ was only detected in one isolate, FZP3105, which also harbors *bla*_CTX-M-65_, *bla*_OXA-1_, and *bla*_OXA-10_. Accordingly, FZP2936, FZP3115, FZP3105, and one *bla*_CTX-M-65_-positive isolate FZP2826 were selected for further analyses by whole-genome sequencing using the Nanopore technology. Three different quinolone resistance genes were present: *aac(6′)-Ib-cr* (14/27, 51.9%), *qnrA1* (2/27, 7.4%), and *qnrD1* (8/27, 29.6%). Aminoglycoside resistance genes were commonly detected in these MDR *Proteus* strains, with *aadA1* (21/27, 77.7%), *aph(3″)-Ib* (17/27, 63.0%), and *aph(6)-Id* (17/27, 63.0%) being the most prevalent. All the isolates, except for FZP1097 and FZP3364, contained both *sul* and *dfrA* genes, conferring co-resistance to sulfamethoxazole-trimethoprim. Albeit only one known resistance gene *hugA* (encoding a class A β-lactamase conferring third-generation cephalosporin-resistance) was detected, FZP1097 was resistant to several antimicrobial agents, such as ciprofloxacin and sulfamethoxazole-trimethoprim in addition to cefotaxime, indicating that some unknown resistance mechanisms may be involved in its MDR phenotype.

### Population structure analysis of MDR *Proteus* isolates

To understand the genetic relationship, the 27 MDR *Proteus* strains were compared to other *Proteus* isolates from different geographic locations in China, and a core genome-based phylogenetic tree was constructed, which revealed two distinct groups (Figure 1). The large group includes all the *P. mirabilis* strains with a high degree of whole-genome homogeneity (0–5,555 SNPs, Supplementary Table S3), and the other one consists of different non-*P. mirabilis* strains of the *Proteus* genus. From the phylogenetic tree, some isolates from different patients are tightly clustered, for example FZP2833/FZP3803 and FZP4349/FZP3364, hinting a common origin and cryptic transmission in this hospital. In addition, clonal relationship was also noticed between some isolates in this study and those from food animals or humans in other locations of China (Supplementary Table S4), for example FZP2958/XH1653 (*P. mirabilis*, humans, Zhejiang of China, 2015) and FZP2095/ZF2 (*P. terrae*, animals, Jiangsu of China, 2018), suggesting the possible circulation and clonal transmission of these MDR *Proteus* strains amongst animals and humans across China. Active monitoring the spread of MDR *Proteus* strains in the context of ‘One Health’ (environmental, animal, and human sectors) is an essential part in combating antimicrobial resistance.

### Genetic features of ICE in MDR *Proteus* isolates

**Figure 2:**
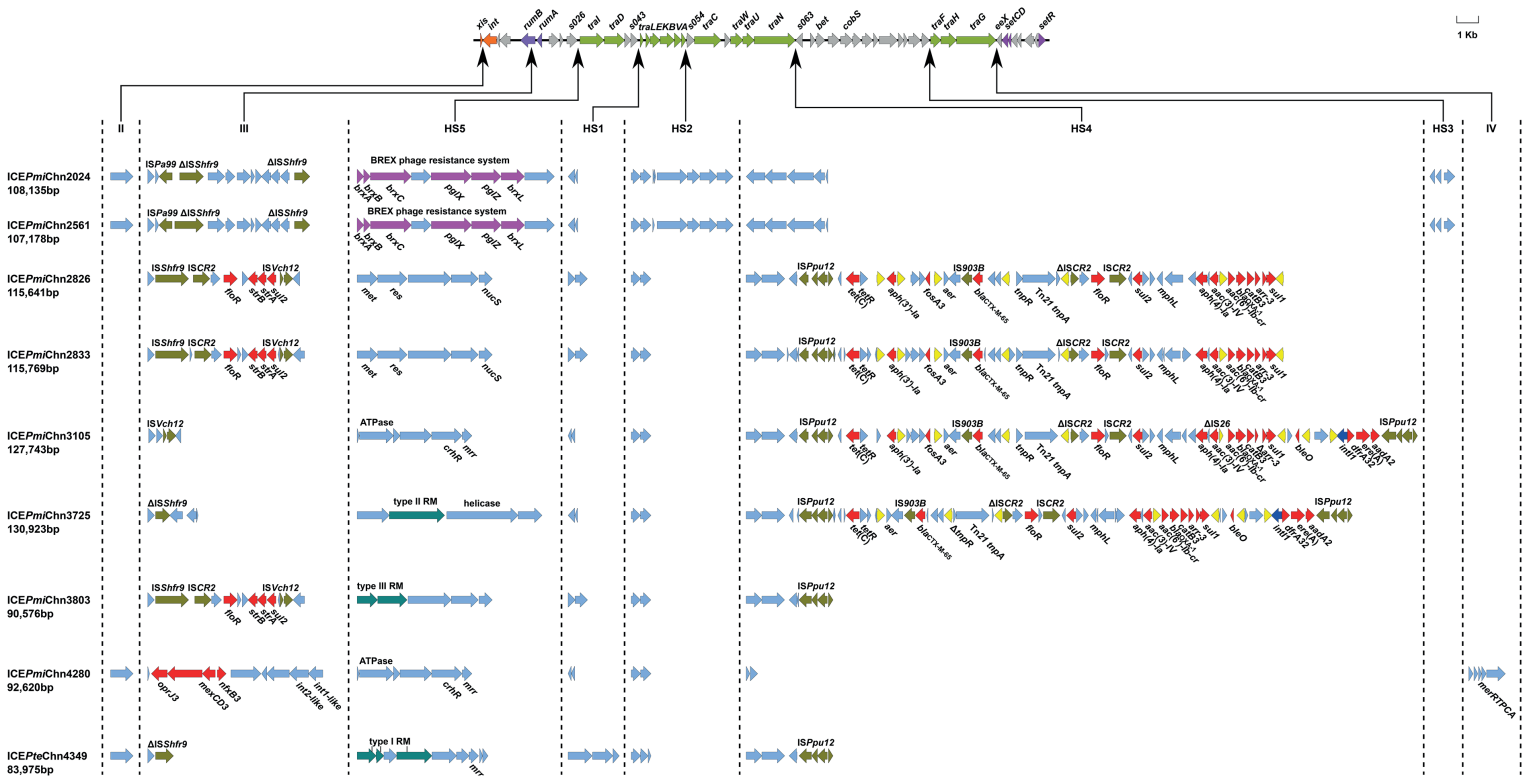
Genetic organization of the ICEs in this study. The upper line shows the backbone of SXT/R391 ICEs with conserved core genes. Arrows in orange, purple, green and amaranth indicate genes involved in site-specific excision and integration (*xis* and *int*), DNA repair (*rumAB*), conjugative transfer (*tra*) and regulation (*setCDR*). Other core genes are indicated by light gray arrows. Under the backbone, hotspots (HS1-HS5) and variable regions (II–IV) are shown, with thin arrows indicating the sites of their insertion. ARGs, insertion sequences, BREX phage resistance system, and restrictive modification systems are highlighted in red, olive, purple-red, and blue-green, respectively, except that IS*26* is highlighted in yellow. Other genes in the insertion regions are indicated by light blue arrows.

Fourteen out of the 27 *Proteus* strains were positive for the *int*_SXT_ gene (> 97% identity to that of SXT), and all of these SXT/R391 ICE-harboring isolates were *P. mirabilis*, except one *P. terrae* (Table 1). Based on the genome data, the complete sequences of nine SXT/R391 ICEs were successfully assembled, ranging in size from 83,975 bp to 130,923 bp, and five were fragmented in two or more contigs (Figure 2). Among them, ICE*Pmi*Chn2826 and ICE*Pmi*Chn2833 are almost identical, with only several different bases in variable regions. ICE*Pmi*Chn2024 and ICE*Pmi*Chn2561 are only differed by an insertion of a 1,063 bp truncated IS*Va2* between *traI* and *traD* in the former.

Analysis of genetic organization showed that the nine ICEs shared a common backbone structure with most SXT/R391 ICEs, but also contained DNA sequences that are relatively specific for individual elements, comprising five hotspots (HS1-5) and three variable regions (VRII, VRIII and VRIV; Figure 2), as described previously (Wozniak et al., 2009). Four ICEs (ICE*Pmi*Chn2024, ICE*Pmi*Chn2561, ICE*Pmi*Chn4280, and ICE*Pte*Chn4349) harbor a *mutL* gene (encoding a putative DNA mismatch repair protein) in the VRII (*xis*-*int*), and ICE*Pmi*Chn4280 solely has a *mer* operon in the VRIV (*traG*-*eex*). All nine ICEs harbored VRIII (disrupting the *rumB* gene), and three ICEs (ICE*Pmi*Chn2826, ICE*Pmi*Chn2833, and ICE*Pmi*Chn3803) have a MDR region bearing ARGs *floR*, *strB/A*, and *sul2*, conferring resistance to chloramphenicol, streptomycin, and sulfamethoxazole, respectively. Specially, ICE*Pmi*Chn4280 has a multi-drug resistance RND efflux pump gene cluster, *tmexCD3*-*toprJ3*, in VRIII (Wang et al., 2021). HS1 (*s043*-*traL*), HS2 (*traA*-*s054*), HS4 (*traN*-*s063*), and HS5 (*s026*-*traI*) were detected in all nine ICEs, while HS3 (*s073*-*traF*) was only found in ICE*Pmi*Chn2024 and ICE*Pmi*Chn2561, with inserted genes encoding an integrase, a dihydrofolate reductase-like protein and a hypothetical protein. Gene clusters encoding diverse restriction-modification (RM) systems conferring resistance to bacteriophages (Balado et al., 2013) are commonly found in HS5 in these ICEs, including the BREX phage resistance system (*brxA-brxB-brxC-pglX-pglZ-brxL*; Slattery et al., 2020). Additionally, genes encoding endonuclease, ATPase, methyltransferase, helicase, and the *mrr* restriction system are also detected in this region (Figure 2).

**Figure 3:**
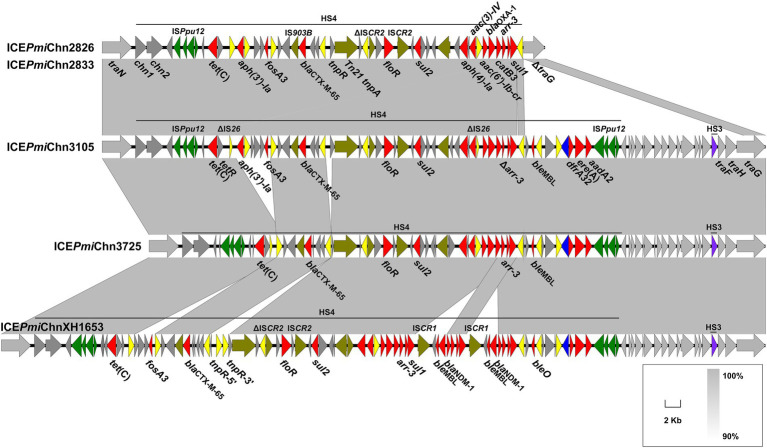
Genetic characteristics of HS4. The HS4-bearing region of ICE*Pmi*Chn2826 (ICE*Pmi*Chn2833), ICE*Pmi*Chn3105, and ICE*Pmi*Chn3725 is compared with that of ICE*Pmi*ChnXH1653 (accession no. CP065039). Genes are indicated by arrows, and those in HS4, HS3 and backbone are indicated in dark grey, purple and light grey, respectively. ARGs, integrase genes, IS*26*, IS*Ppu12*, and other transposase genes are highlighted in red, blue, yellow, green, and olive, respectively. Shared regions of > 90% nucleotide sequence identity are indicated by grey shading.

Abundant ARGs are present in HS4 in ICE*Pmi*Chn2826, ICE*Pmi*Chn2833, ICE*Pmi*Chn3105, and ICE*Pmi*Chn3725. Structural comparison showed that the four ICEs share similar gene contents in the HS4 region, which is also similar to that in ICE*Pmi*ChnXH1653 detected in a *P. mirabilis* strain from a urine sample of a patient in Zhejiang, China, in 2015 (He et al., 2021; Figure 3), indicating a common origin. Like the scenario in ICE*Pmi*ChnXH1653, the HS4 in ICE*Pmi*Chn3105 and ICE*Pmi*Chn3725 are also present as an IS*Ppu12*-mediated region, but the two tandem copies of a *bla*_NDM-1_-bearing IS*CR1* element downstream of *sul1*, and two copies of IS*26* interrupting *tnpR* are not detected. It has been known that a second IS*26* preferentially inserted into adjacent positions of the existing IS*26*, and the recombination between the two copies of IS26 would cause deletion, insertion or translocation (Partridge et al., 2018). The HS4 in ICE*Pmi*Chn2826 (ICE*Pmi*Chn2833) seems to be a variant of ICE*Pmi*Chn3105 with the deletion of 9,781 bp HS4 region and 15,456 bp of adjacent backbone, very likely resulting from an insertion of the second IS*26* inside the *traG* and the subsequent recombination action of IS*26* (Figure 3). The HS4 in ICE*Pmi*Chn3105 and ICE*Pmi*Chn3725 are highly similar (99.97% nucleotide identity at 92% coverage), and an IS*26-*mediated excision of a 4,311 bp region bearing *aph(3′)-Ia* and *fosA3* downstream of *tetR*, and an 813 bp deletion upstream of *tnpR* represent two major modular differences of them (Figure 3). This finding suggests that these MDR ICEs are undergoing rapid evolution within healthcare environments.

**Figure 4:**
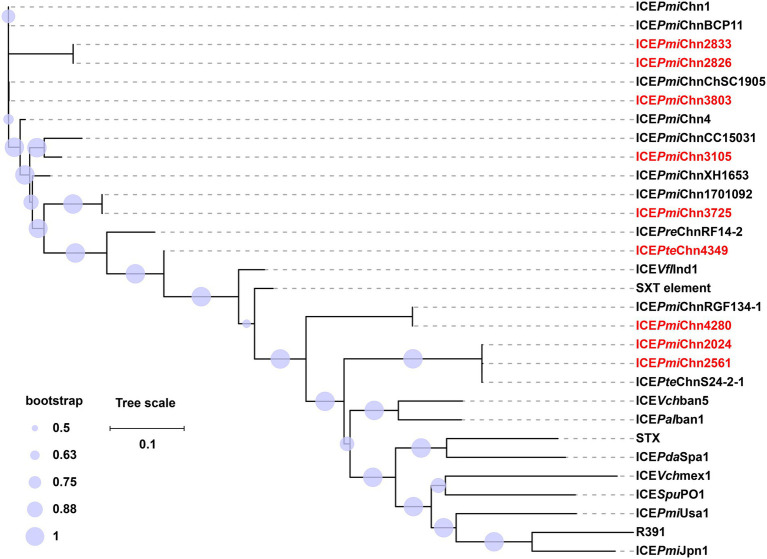
Phylogenetic analysis of ICEs in this study and 21 representative SXT/R391 ICEs. The phylogenetic tree of ICEs was constructed based on the SNPs in their conserved regions with the maximum-likelihood method in FastTree. ICEs in this study are highlighted in red. The tree scale indicates substitutions per site. Detailed information of these ICEs is presented in Supplementary Table S6.

By BLASTn analysis, the nine SXT/R391 ICEs exhibited similarity to many different ICEs recovering from food animals and humans in different locations of China (Supplementary Table S5), indicating wide spread of them. The core gene phylogenetic analysis showed that ICE*Pmi*Chn2826, ICE*Pmi*Chn2833, and ICE*Pmi*Chn3803 are closely related to the reference MDR ICEs, ICEPmiChn1 (*P. mirabilis*, chicken, Hubei of China), ICEPmiChnBCP11 (*P. mirabilis*, swine, Sichuan of China), and ICEPmiChnChSC1905 (*P. mirabilis*, swine, Sichuan of China; Figure 4; Supplementary Table S6). ICE*Pmi*Chn3105 is clustered with ICE*Pmi*ChnCC15031 that was detected in a *P. mirabilis* strain from a dog in Jilin, China. ICE*Pmi*Chn3725 is closely related, with only 1 SNP, to ICEPmiChn1701092 from a *P. mirabilis* strain recovered from the intestinal contents of humans in Zhejiang, China. The phylogenetic analysis indicated that ICE*Pte*Chn4349 serves as a potentially novel SXT/R391 element as it forms a distinct clade separated from other representative ICEs (Figure 4; Supplementary Table S6). ICE*Pmi*Chn2024, ICE*Pmi*Chn2561, and ICE*Pmi*Chn4280 are more distantly related to the ICEs in this study. ICE*Pmi*Chn4280 has the most closely genetic relationship with ICEPmiChnRGF134-1 (*P. mirabilis*, swine, Jiangsu of China), and ICE*Pmi*Chn2024 (ICE*Pmi*Chn2561) is closest to ICEPteChnS24-2-1 (*P. terrae*, Cacatua, Guangzhou of China), with 0 and 2 SNPs, respectively (Figure 4; Supplementary Table S6). These findings suggest the cryptic dissemination of these ICEs in *Proteus* strains on a China-wide scale.

According to the species tree (Figure 1), we found that strains FZP2024 and FZP2561 are little related, but share almost identical ICEs (ICE*Pmi*Chn2024 and ICE*Pmi*Chn2561). The closely related strains FZP2826 and FZP3803 bear ICEs (ICE*Pmi*Chn2826 and ICE*Pmi*Chn3803) with markedly different HS4 and HS5 contents. Besides, strains FZP4349 and FZP3364 are tightly clustered, and the latter is devoid of an SXT/R391 element on the chromosome while the former possesses the ICE*Pte*Chn4349. These findings suggest the independent acquisition and horizontal transmission of SXT/R391 ICEs across the *Proteus* population (Sato et al., 2020).

We also detected the circular intermediate of ICEs by using the primers LE4 and RE4 as previously described (Lei et al., 2016). Results showed that the circular form of ICEs could be detected in all 14 ICE-harboring strains, revealing a potential transmission pattern of them. To determine the transfer ability of these ICEs, we selected three ICE-carrying isolates (FZP2826, FZP3725 and FZP4280) for the conjugation experiments. Results showed that ICE*Pmi*Chn3725 could be transferred to *E. coli* J53 at a frequency of ~9 × 10^−5^ transconjugants per recipient cell. Antimicrobial susceptibility analysis showed that the acquisition of ICE*Pmi*Chn3725 enables *E. coli* J53 to become resistant to gentamicin, cefotaxime, aztreonam, ampicillin/sulbactam, and trimethoprim/sulfamethoxazole (Supplementary Table S7), despite that resistance to aztreonam conferred by CTX-M-65 in FZP3725 remains within the sensitivity range. We hypothesize that the hydrolysis of aztreonam by CTX-M-65 is possibly inhibited in FZP3725 by an unknown regulatory mechanism, but it works normally in *E. coli* J53. The conjugation of ICE*Pmi*Chn2826 and ICE*Pmi*Chn4280 were failed despite repeated attempts, suggesting that both ICEs are not self-transmissible. The non-transferability of ICE*Pmi*Chn2826 may be caused by the truncated *traG,* and the deleted *traF* and *traH* (Figure 3).

### Genetic features of the *bla*_NDM-1_-harboring plasmid pNDM_FZP3105

**Figure 5:**
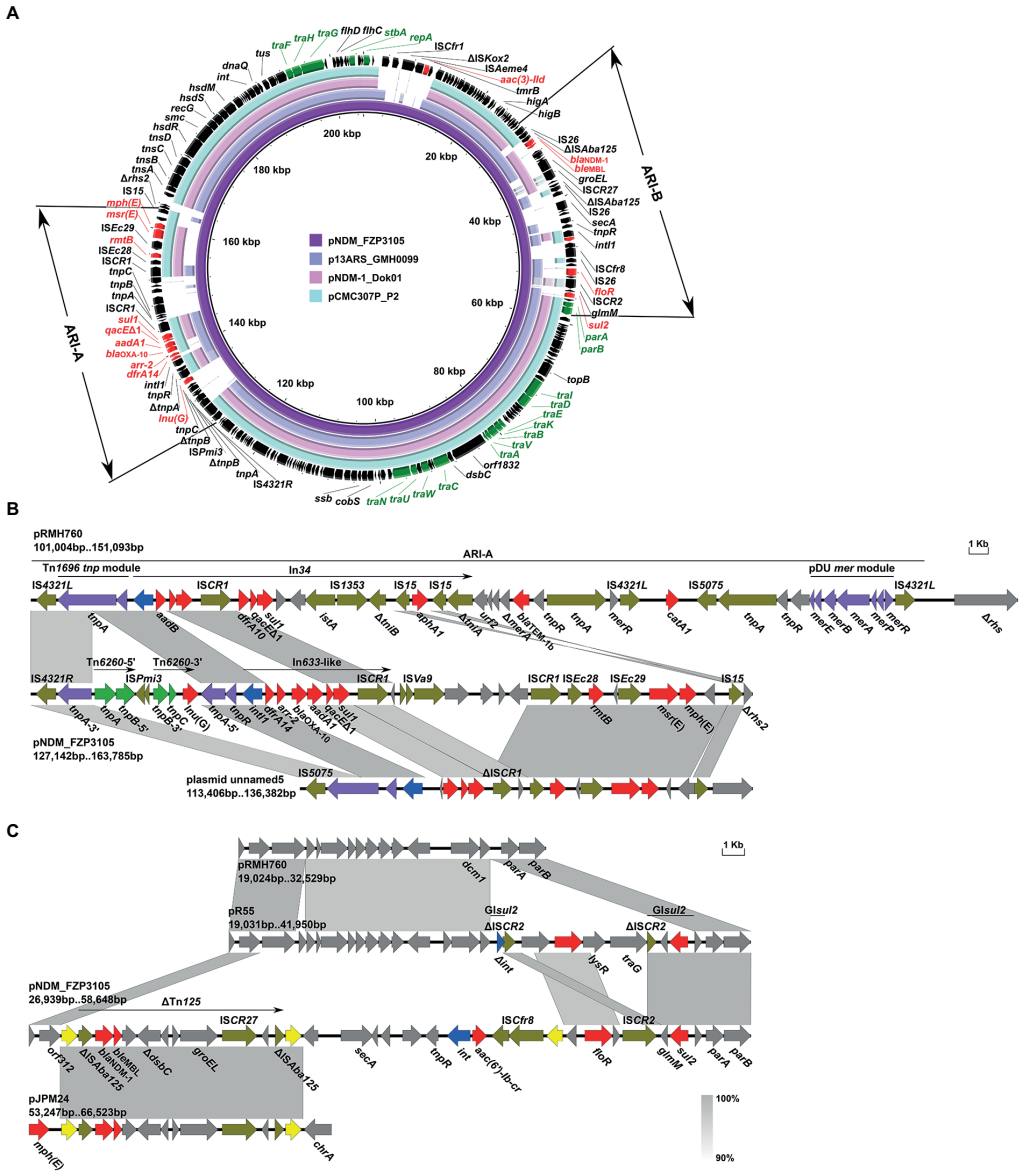
Genetic features of pNDM_FZP3105. **(A)** Circular comparison of pNDM_FZP3105 with pCMC307P_P2, p13ARS_GMH0099 and pNDM-1_Dok01. Arrows on the outer ring indicate deduced ORFs and their orientations. Genes for replication (*repA*), conjugal transfer (*tra*), and plasmid maintenance (*stbA*, *parAB*) are highlighted in green. ARGs are highlighted in red. Two antibiotic resistance islands, ARI-A and ARI-B, are indicated. **(B)** Organization of the ARI-A of pNDM_FZP3105, and comparisons to related regions. **(C)** Organization of the ARI-B of pNDM_FZP3105, and comparisons to related regions. Genes are denoted by arrows, and ARGs, integrase genes, IS*26*, and other transposase genes are highlighted in red, blue, yellow, and olive, respectively. The Tn*1696 tnp* and pDU *mer* modules are indicated in purple, and Tn6260 is in green. Regions of >90% identity are indicated by grey shading. Δ represents truncated genes.

*P. mirabilis* FZP2937 and FZP3105 were recovered from the same patient, and were sampled 18 days apart, from wound secretion and sanies, respectively. FZP2937 and FZP3105 are clonally related as they share identical core genomes (0 SNP). Despite that, the two strains exhibited different phenotypic resistance (Table 1), and the most noteworthy feature is that FZP3105 shows resistant to meropenem and ceftazidime, to which FZP2937 is susceptible. Genome data revealed that the later strain FZP3105 additionally acquired an NDM-1-producing MDR plasmid pNDM_FZP3105. This plasmid is 205,118 bp in size, and belongs to IncC ST3 (*A053*-*parA*-*parB*-*repA* allele number 1–2–2-2) incompatibility group. BLASTn analysis showed that it has >99.8% nucleotide identity (≥77% coverage) to pCMC307P_P2 (CP079626, *Klebsiella pneumoniae*, India), p13ARS_GMH0099 (LR697099, *K. pneumoniae*, United Kingdom), and pNDM-1_Dok01 (AP012208, *E. coli*, Japan; Figure 5A). pNDM_FZP3105 is a type 1/2 hybrid IncC plasmid, as it contains *orf1832* (characteristic of type 1) and Δ*rhs2* (characteristic of type 2), lacking of two additional sequences i1 and i2 (Harmer and Hall, 2014). The backbone of pNDM_FZP3105 is 112,618 bp in size with an average 51.05% G + C content. Nucleotide (nt) 56,193–126,943 of pNDM_FZP3105 showed 99.94% identity to that of type 1 IncC reference plasmid pR148 (JX141473, *Aeromonas hydrophila*, South Korea), and nt 1–2,522, 11,597–28,308, 163,334–165,885, and 185,018–205,098 showed 99.98% identity to that of type 2 IncC reference plasmid pR55 (JQ010984, *K. pneumoniae*, France; Supplementary Figure S1). Conjugation assays showed that pNDM_FZP3105 was able to conjugate into *E. coli* EC600, and the acquisition of pNDM_FZP3105 greatly increased meropenem resistance in EC600 by at least 8-fold (Supplementary Table S8).

pNDM_FZP3105 carries three accessory modules, namely the antibiotic resistance island (ARI)-A, ARI-B, and the IS*Cfr1*-*aac(3)*-*IId-tmrB* module. ARI-A (nt 127,160 to 163,333) is located immediately upstream of the 452 bp *rhs2* remnant (Figure 5B). Compared to the prototype ARI-A of a complex transposon structure bounded by 38-bp inverted repeats (IRs) of Tn*1696 tnp* and pDU *mer* modules interrupted by either IS*4321* or IS*5075* (Harmer and Hall, 2015), like the case in pRMH760 (KF976462, *K. pneumoniae*, Australia), ARI-A in pNDM_FZP3105 has only retained the IS*4321*-*tnpAR* structure, though it is interrupted by the Tn*6260* that is truncated by an IS*Pmi3* inside of *tnpA*. Instead of In*34* in ARI-A in pRMH760, an ΔIn*633*-like segment *intI1*-*dfrA14*-*arr-2*-*bla*_OXA-10_-*aadA1*-*qacEΔ1*-*sul1*-IS*CR1* was present in pNDM_FZP3105, followed by a 6.5-kb region carrying an IS*Va9*, a DNA repair ATPase and three hypothetical proteins, and a 10.7-kb module IS*CR1*-IS*Ec28*-*rmtB*-IS*Ec29*-*msr*(E)-*mph*(E)-IS*15*. BLASTn analysis showed that ARI-A in pNDM_FZP3105 has 99.9% nucleotide identity at 59% coverage to plasmid unnamed5 (CP029118, *E. coli*, United States), but with genetic element insertions, deletions, and replacements (Figure 5B). These findings suggest that ARI-A in pNDM_FZP3105 is a novel MDR mosaic region. *Lnu*(G), which confers resistance to lincomycin by nucleotidylation, is commonly found on the chromosomes of Enterococcus, and also on the plasmids containing replicons of IncFIA(HI1), IncHI1A and IncHI1B(R27), or in combination with IncFII or IncX4 in Enterobacteriaceae (Li et al., 2021). To our knowledge, this is the first report of *lnu*(G) in an IncC plasmid.

*bla*_NDM_ was found within ARI-B (nt 28,372–55,887) in pNDM_FZP3105, but not in ARI-A module as usually reported (Harmer and Hall, 2014; Wailan et al., 2016). When compared to pR55, ARI-B in pNDM_FZP3105 has gained two IS*26-*bracketed segments, a 9.0 kb *bla*_NDM-1_-carrying ΔTn*125* and a 10.6 kb *aac(6′)-Ib-cr*-carrying region, causing the deletion of a 9.7 kb of the A/C2 backbone (immediately downstream of *orf312*), and the *int* end of GI*sul2*, but has retained a 2.4 kb *floR*-containing fragment and the last 2.8 kb of the *sul2* end of GI*sul2* (Figure 5C). Especially, the two copies of IS*26* surrounding ΔTn*125* have the potential to form a composite transposon to mobilize the intervening genetic components including *bla*_NDM-1_. However, no 8-bp DRs were identified, indicating the occurrence of homologous recombination. Similar (>99.8% identity) IS*26*-ΔTn*125*-IS*26* unit was also found on several *Proteus* chromosomes and plasmids, suggesting an important role of IS*26* in the spread of *bla*_NDM-1_ in *Proteus* species.

The IS*Cfr1-aac(3)-IId*-*tmrB* module, with the genetic structure *orf378*-IS*Cfr1*-ΔIS*Kox2*-IS*Aeme4*-*aac(3)-IId*-*tmrB*, is located 1,457 bp downstream of *repA*, breaking and truncating the *orf190* gene (encoding a hypothetical protein) downstream of *cysH* (Supplementary Figure S1). An insertion in this location is barely seen in IncC plasmids, and similar (>99% coverage, >99% identity) *aac(3)-IId*-*tmrB* module is only identified in pVFN3-blaOXA-193 K (CP089604, *Vibrio furnissii*, hospital sewage, China) by BLASTn analysis. The mobilization mechanism of the IS*Cfr1*-*aac(3)-IId*-*tmrB* module remains unknown.

### Characteristics of *bla*_KPC-2_-carrying *Proteus mirabilis* FZP2936 and FZP3115

FZP2936 and FZP3115 are two subsequent isolates from a single patient, from urine and wound secretion, respectively, 19 days apart. The two strains share identical core genomes as well as resistance profiles, showing the clonal nature of them. FZP2936 and FZP3115 both have a 4,245,458-bp circular chromosome and two closed plasmids. 15 ARGs were identified in both strains, and all of them, except *bla*_KPC-2_, are located on the chromosome. *bla*_KPC-2_ is present on 24,225-bp IncN plasmids pKPC_FZP2936 and pKPC_FZP3115, which are identical to the previously reported pT211(CP017083) from a clinical *P. mirabilis* strain T21 in 2013, in Zhejiang province (approximately 1,830 km apart in distance from Sichuan), China (Hua et al., 2020). The finding suggests the likelihood of a wide dissemination of the pT211-like *bla*_KPC-2_-bearing plasmids in *proteus* in China, which warrant more surveillance. Given that FZP2936 and FZP3115 have close genetical relationship to strain T21 (7 SNPs; Figure 1), and that pT211 is not self-transferable as previously revealed (Hua et al., 2020), the diffusion of pT211-like plasmids is likely due to expansion of bacterial clones.

**Figure 6:**
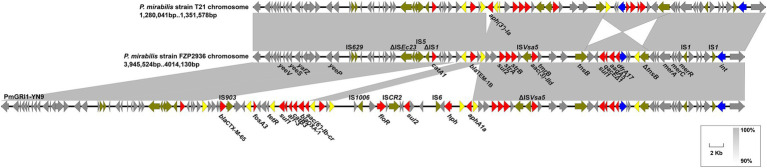
Genetic features of the multidrug-resistant genomic island in FZP2936. The genomic island in FZP2936 is compared with PmGRI1-YN9 and related region in *Proteus mirabilis* strain T21. Genes are denoted by arrows, and ARGs, integrase genes, IS*26*, and other transposase genes are highlighted in red, blue, yellow, and olive, respectively. Regions of > 90% identity are indicated by grey shading. Δ represents truncated genes.

The *dfrA1*-*sat2*-*aadA1* gene cassettes in both strains are included in the Tn*7*-like transposon 20 bp downstream of the *glmS* gene on the chromosome, as described in *P. mirabilis* strain T21 (Hua et al., 2020). The remainder of the chromosomal ARGs were mainly clustered in a 64,829 bp MDR genomic island PmGRI1 (Lei et al., 2020), which was later identified as a variant of PmGRI1-YN9 (Ma et al., 2021; MW699445, *P. mirabilis*, animal, China) by BLASTn analysis. Compared to the configuration of PmGRI1-YN9, the PmGRI1 in FZP2936 (FZP3115) have two IS*26*-mediated excision situating 1,339 bp upstream and 533 bp downstream of *bla*_TEM-1B_, with 14,053 bp and 20,022 bp in size, respectively, resulting in the loss of several ARGs (Figure 6). Besides, the PmGRI1 in this study was almost identical to that in *P. mirabilis* strain T21, except an IS*26*-mediated deletion of *aph(3′)-Ia* 1,491 bp downstream of *bla*_TEM-1B_, and a *tnsB*-mediated reversal of a 7,275 bp region bearing a class I integron with the cassette array *dfrA17*-*addA5*-*qacEΔ1*-*sul1* (Figure 6). These results highlight the high plasticity of PmGRI1, and also reinforce the great ability of IS*26* to accumulate ARGs, and to promote the diversity of the multidrug resistance regions in mobile genetic elements (He et al., 2021).

PmGRI1 is a new GI carrying various resistance genes initially identified in a *P. mirabilis* strain from chicken in 2018 (Lei et al., 2020), and was later detected in several *P. mirabilis* isolates of swine and chicken origins in different locations of China (Ma et al., 2021). To determine the prevalence of PmGRI1 in *Proteus* isolates in the same hospital, we detected the PmGRI1 by searching the integrase gene *int* in the remaining 25 strains, and found that 10 of them were positive for the PmGRI1 integrase gene (Table 1). This finding indicates the possible prevalence of PmGRI1 variants in *Proteus* strains in both the animals and humans. More data is needed to better understand the epidemiology and dynamic evolution of PmGRI1 in China.

## Conclusion

In this study, we demonstrated the genetic diversity of MDR *Proteus* strains and spotlighted the important roles of GIs, especially SXT/R391 ICEs, and plasmids in capturing and spreading ARGs in *Proteus*. Our study also raises concern that these MDR *Proteus* strains might be circulating and undergoing rapid evolution amongst animals and humans across China. From ‘One Health’ perspective, active surveillance for MDR *Proteus* in the environment is urgently needed.

## Data availability statement

The datasets presented in this study can be found in online repositories. The names of the repository/repositories and accession number(s) can be found in the article/Supplementary material.

## Author contributions

YL: conceptualization, formal analysis, and writing-original draft. QL, YQ, and HC: methodology, resources, and formal analysis. XD, CF, and LZ: methodology and software. YZ and JS: writing-review and editing. LZ: conceptualization, writing-review and editing, and supervision. All authors contributed to the article and approved the submitted version.

## Funding

This work was supported by National Natural Science Foundation of China (31900125), Scientific and technological project in Sichuan Province (2022JDRC0144), the Joint Funds of the Luzhou and Southwest Medical University Natural Science Foundation (2019LZXNYDJ47 and 2020LZXNYDJ34), and Central Government Funds for Guiding Local Scientific and Technological Development of Sichuan Province (2021ZYD0084). The funders had no role in study design, data collection and interpretation, or the decision to submit the work for publication.

## Conflict of interest

The authors declare that the research was conducted in the absence of any commercial or financial relationships that could be construed as a potential conflict of interest.

## Publisher’s note

All claims expressed in this article are solely those of the authors and do not necessarily represent those of their affiliated organizations, or those of the publisher, the editors and the reviewers. Any product that may be evaluated in this article, or claim that may be made by its manufacturer, is not guaranteed or endorsed by the publisher.

## Footnotes

^1^ https://jspecies.ribohost.com/jspeciesws/#analyse

^2^ https://itol.embl.de/
