# Supplementary material for: Genomic characteristics of clinical multidrug-resistant Proteus isolates from a tertiary care hospital in southwest China
Source: Front Microbiol. 2022 Aug 24;13:977356. doi: 10.3389/fmicb.2022.977356 (PMC9449695; doi:10.3389/fmicb.2022.977356)
Supplement: Supplementary file 1 [file Table_1.docx]

Supplementary Material

**Table S1 List of antibiotics used in the Antibiotic susceptibility testing.**

| **Class** | **Drugs** |
| --- | --- |
| β-lactams | AMP, ampicillin；ATM, aztreonam; CAZ, ceftazidime; CEP, cefalotin; CPD, cefpodoxime; CRO, ceftriaxone; CTT, cefotetan; CTX, cefotaxime; CXA, cefuroxime axetil; CXM, cefuroxime; CZO, cefazolin; CZX, ceftizoxime; ETP, ertapenem; FEP, cefepime; FOX, cefoxitin; IPM, imipenem; MEM, meropenem; PIP, piperacillin |
| β-lactams/β-lactamase inhibitors | AMC, amoxicillin/clavulanic acid; CSL, cefoperazone/sulbactam; SAM, ampicillin/sulbactam; TZP, piperacillin/tazobactam |
| quinolones | LVX, levofloxacin; MFX, moxifloxacin; NOR, norfloxacin; CIP, ciprofloxacin |
| aminoglycosides | AMK, amikacin; GEN, gentamicin; TOB, tobramycin |
| sulfonamides | SXT, trimethoprim/sulfamethoxazole |
| tetracyclines | TCY, tetracycline; TGC, tigecycline |
| nitrofurans | NIT, nitrofurantoin |

**Table S2 General features of the genomes of *proteus* strains.**

| **Strain** | **Clean reads (Gb)** | **Draft genome size (bp)** | **GC content** | **No. of contigs** | **No. of contigs ≥ 1,000 bp** | **GenBank**  **accession no.** |
| --- | --- | --- | --- | --- | --- | --- |
| FZP1665 | 1.59 | 4,022,129 | 38.95 | 41 | 33 | JAMOBO000000000 |
| FZP2056 | 1.44 | 3,987,527 | 38.9 | 47 | 37 | JAMOBP000000000 |
| FZP2095 | 1.37 | 3,990,371 | 37.94 | 22 | 16 | JAMQBJ000000000 |
| FZP2128 | 1.44 | 4,005,233 | 38.94 | 48 | 39 | JAMOJB000000000 |
| FZP2958 | 1.31 | 4,004,376 | 39.0 | 41 | 32 | JAMOJC000000000 |
| FZP3803 | 1.52 | 3,974,993 | 38.9 | 54 | 43 | JAMOJD000000000 |
| FZP4264 | 1.45 | 3,981,522 | 38.83 | 54 | 47 | JAMOJE000000000 |
| FZP4280 | 1.78 | 4,099,380 | 39.13 | 55 | 42 | JAMOJR000000000 |
| FZP4349 | 1.46 | 4,196,176 | 38.24 | 48 | 40 | JAMOJS000000000 |
| FZP4423 | 1.61 | 4,072,591 | 39.06 | 52 | 40 | JAMOJT000000000 |
| FZP4515 | 1.62 | 3,890,653 | 38.8 | 52 | 42 | JAMOJU000000000 |
| FZP1097 | 1.36 | 3,766,980 | 37.68 | 45 | 36 | JAMOJQ000000000 |
| FZP1177 | 1.12 | 3,929,510 | 38.81 | 44 | 34 | JAMOJF000000000 |
| FZP1611 | 1.26 | 3972625 | 38.77 | 50 | 39 | JAMOJG000000000 |
| FZP2024 | 1.23 | 4,100,034 | 39.1 | 52 | 42 | JAMOJH000000000 |
| FZP2561 | 1.09 | 3,895,621 | 38.83 | 37 | 31 | JAMOJI000000000 |
| FZP2833 | 0.95 | 3,972,123 | 38.83 | 53 | 39 | JAMOJJ000000000 |
| FZP2937 | 1.37 | 4,054,355 | 38.96 | 50 | 40 | JAMOJK000000000 |
| FZP3043 | 1.48 | 3,981,832 | 38.92 | 50 | 39 | JAMOJL000000000 |
| FZP3320 | 1.44 | 4,063,293 | 38.78 | 31 | 24 | JAMOJM000000000 |
| FZP3364 | 1.30 | 3,871,523 | 37.92 | 22 | 21 | JAMOJN000000000 |
| FZP3406 | 1.27 | 4,151,858 | 38.93 | 45 | 38 | JAMOJO000000000 |
| FZP3725 | 1.08 | 4,103,770 | 39.05 | 58 | 46 | JAMOJP000000000 |
| FZP2826 | 1.36 | 4,026,988 | 38.99 | 1 | 1 | CP098446 |
| FZP2936 | 1.44 | 4,245,458 | 39.12 | 3 | 3 | CP098447 |
| FZP3115 | 1.44 | 4,245,458 | 39.12 | 3 | 3 | CP098450 |
| FZP3105 | 0.93 | 4,312,705 | 39.67 | 2 | 2 | CP098444 |

**Table S4 Detailed information of *Proteus* strains (from the literature) used in the phylogenetic tree.**

| **Name** | **Species** | **Source** | **Locations of China** | **Year** | **Accession no.** |
| --- | --- | --- | --- | --- | --- |
| BC11-24 | *P. mirabilis* | Animals | Sichuan | 2016 | CP026571 |
| C55 | *P. mirabilis* | Animals | Shandong | 2018 | CP044436 |
| C74 | *P. mirabilis* | Animals | Shandong | 2018 | CP044437 |
| JPM24 | *P. mirabilis* | Animals | Guangdong | 2017 | CP053894 |
| NMG38-2 | *Proteus sp.* | Animals | Inner Mongolia | 2018 | CP085481 |
| PmSC1111 | *P. mirabilis* | Animals | Sichuan | 2017 | CP034090 |
| RGF134-1 | *P. mirabilis* | Animals | Jiangsu | 2019 | CP066833 |
| SNYG35 | *P. mirabilis* | Animals | Sichuan | 2018 | CP047589 |
| T18 | *P. mirabilis* | Humans | Zhejiang | 2014 | CP017085 |
| T21 | *P. mirabilis* | Humans | Zhejiang | 2013 | CP017082 |
| T60 | *Proteus columbae* | Animals | Henan | 2019 | CP043925 |
| XH1653 | *P. mirabilis* | Humans | Zhejiang | 2015 | CP065039 |
| YPM35 | *P. mirabilis* | Animals | Guangdong | 2017 | CP053898 |
| ZF2 | *Proteus terrae* | Animals | Jiangsu | 2018 | CP045008 |

**Table S5 Information of the top three alignment results for each ICE we analyzed.**

| **ICE name** | **Similar ICEs from species** | **Query cover (%) / Identity (%)** | **Source** | **Country** | **Accession no.** |
| --- | --- | --- | --- | --- | --- |
| ICE*Pmi*Chn2024 | *Proteus mirabilis* strain MPE5139 | 100/99.99 | Humans | China | CP053684.1 |
|  | *Proteus terrae* subsp. *cibarius* strain S24-2-1 | 99/99.99 | Animals | China | CP073630.1 |
|  | *Proteus mirabilis* strain TJ3300 | 98/99.99 | Humans | China | KX243415.1 |
| ICE*Pmi*Chn2561 | *Proteus terrae* subsp. *cibarius* strain S24-2-1 | 100/99.99 | Animals | China | CP073630.1 |
|  | *Proteus mirabilis* strain MPE5139 | 100/99.97 | Humans | China | CP053684.1 |
|  | *Proteus mirabilis* strain TJ3300 | 99/99.98 | Humans | China | KX243415.1 |
| ICE*Pmi*Chn2826 | *Proteus mirabilis* strain H63 | 99/100 | Humans | China | OL790811.1 |
|  | *Klebsiella pneumoniae* strain QD23 | 99/100 | Humans | China | CP042858.1 |
|  | *Proteus mirabilis* strain Yak 2019 | 99/99.99 | Humans | China | CP063440.1 |
| ICE*Pmi*Chn2833 | *Proteus mirabilis* strain H63 | 99/100 | Humans | China | OL790811.1 |
|  | *Klebsiella pneumoniae* strain QD23 | 99/100 | Humans | China | CP042858.1 |
|  | *Proteus mirabilis* strain Yak 2019 | 99/99.99 | Humans | China | CP063440.1 |
| ICE*Pmi*Chn3105 | *Proteus mirabilis* strain CC15031 | 95/99.97 | Animals | China | CP048787.1 |
|  | *Proteus mirabilis* strain XH1653 | 90/99.98 | Humans | China | CP065039.2 |
|  | *Proteus mirabilis* strain S012 | 90/99.97 | Animals | China | CP062146.1 |
| ICE*Pmi*Chn3725 | *Proteus mirabilis* strain 1701092 | 100/99.98 | Humans | China | CP042857.1 |
|  | *Proteus mirabilis* strain XH1653 | 86/99.97 | Humans | China | CP065039.2 |
|  | *Proteus mirabilis* strain S012 | 86/99.97 | Animals | China | CP062146.1 |
| ICE*Pmi*Chn3803 | *Proteus mirabilis* strain ChSC1905 | 100/99.98 | Animals | China | CP047929.1 |
|  | *Proteus mirabilis* ICEPmiChn1 | 100/99.97 | Animals | China | KT962845.1 |
|  | *Proteus mirabilis* ICEPmiChn-BCP11 | 100/99.97 | Animals | China | MG773277.1 |
| ICE*Pmi*Chn4280 | *Proteus mirabilis* strain RGF134-1 | 97/100 | Animals | China | CP066833.1 |
|  | *Proteus mirabilis* ICEPmiChn4 | 73/97.59 | Animals | China | KY437728.1 |
|  | *Proteus mirabilis* strain CC15031 | 71/98.26 | Animals | China | CP048787.1 |
| ICE*Pte*Chn4349 | *Providencia rettgeri* strain PROV002 | 94/97.75 | Humans | China | CP059345.1 |
|  | *Providencia rettgeri* ICEPreChnRF14-2 | 92/99.97 | Animals | China | MT219827.1 |
|  | *Proteus mirabilis* strain MD20140904 | 91/99.99 | Humans | China | KX243411.1 |

**Table S6** **Detailed information of ICEs used in the phylogenetic tree.**

| **Name** | **Species** | **Isolation source** | **Location** | **Year** | **Accession no.** |
| --- | --- | --- | --- | --- | --- |
| SXT | *Vibrio cholerae O139* | Unknown | India | 2002 | AY055428 |
| R391 | *Providencia rettgeri* | Unknown | South Africa | 1967 | AY090559 |
| ICE*Pmi*ChnRGF134-1 | *Proteus mirabilis* | Animals | Jiangsu, China | 2019 | CP066833 |
| ICE*Pmi*Jpn1 | *Proteus mirabilis* | Animals | Sichuan, China | 2015 | KT894734 |
| ICE*Pmi*Usa1 | *Proteus mirabilis* | Unknown | United States | 1986 | AM942759 |
| ICE*Spu*PO1 | *Shewanella putrefaciens* | Marine sediments | Pacific Ocean | 2000 | CP000503 |
| ICE*Vch*mex1 | *Vibrio cholerae non O1-O139* | Unknown | Mexico | 2001 | GQ463143 |
| STX | *Vibrio cholerae* | Unknown | United States | 2001 | AY055428 |
| ICE*Pda*Spa1 | *Photobacterium damselae* | Solea senegalensis | Spain | 2003 | AJ870986 |
| ICE*Pal*ban1 | *Providencia alcalifaciens* | Unknown | Bangladesh | 1999 | GQ463139 |
| ICE*Vch*ban5 | *Vibrio cholerae O1* | Unknown | Bangladesh | 1998 | GQ463140 |
| ICE*Pte*ChnS24-2-1 | *Proteus terrae* | Animals | Guangzhou, China | 2021 | CP073630 |
| ICE*Vfl*Ind1 | *Vibrio fluvialis* | Unknown | India | 2002 | GQ463144 |
| ICE*Pre*ChnRF14-2 | *Providencia rettgeri* | Animals | Jiangsu, China | 2019 | MT219827 |
| ICE*Pmi*Chn1701092 | *Proteus mirabilis* | Humans | Zhejiang, China | 2017 | CP042857 |
| ICE*Pmi*ChnXH1653 | *Proteus mirabilis* | Humans | Zhejiang, China | 2015 | CP065039 |
| ICE*Pmi*ChnCC15031 | *Proteus mirabilis* | Animals | Jilin, China | 2020 | CP048787 |
| ICE*Pmi*Chn4 | *Proteus mirabilis* | Animals | Shandong, China | 2017 | KY437728 |
| ICE*Pmi*ChnChSC1905 | *Proteus mirabilis* | Animals | Sichuan, China | 2020 | CP047929 |
| ICE*Pmi*ChnBCP11 | *Proteus mirabilis* | Animals | Sichuan, China | 2016 | MG773277 |
| ICE*Pmi*Chn1 | *Proteus mirabilis* | Animals | Hubei, China | 2013 | KT962845 |
| ICE*Pmi*Chn2024 | *Proteus mirabilis* | Humans | Sichuan, China | 2021 | This study |
| ICE*Pmi*Chn2561 | *Proteus mirabilis* | Humans | Sichuan, China | 2021 | This study |
| ICE*Pmi*Chn2826 | *Proteus mirabilis* | Humans | Sichuan, China | 2021 | This study |
| ICE*Pmi*Chn2833 | *Proteus mirabilis* | Humans | Sichuan, China | 2021 | This study |
| ICE*Pmi*Chn3105 | *Proteus mirabilis* | Humans | Sichuan, China | 2021 | This study |
| ICE*Pmi*Chn3725 | *Proteus mirabilis* | Humans | Sichuan, China | 2021 | This study |
| ICE*Pmi*Chn3803 | *Proteus mirabilis* | Humans | Sichuan, China | 2021 | This study |
| ICE*Pmi*Chn4280 | *Proteus mirabilis* | Humans | Sichuan, China | 2021 | This study |
| ICE*Pte*Chn4349 | *Proteus terrae* | Humans | Sichuan, China | 2021 | This study |

**Table S7 MICs for FZP3725, its transformant and the recipient strain *E. coli* J53.**

| **Strains** | **MIC (μg/ml) ^a^** | | | | | | | | | |  |
| --- | --- | --- | --- | --- | --- | --- | --- | --- | --- | --- | --- |
|  | **AMK** | **GEN** | **SXT** | **MEM** | **IMP** | **CAZ** | **SAM** | **CIP** | **CTX** | **ATM** | |
| FZP3725 | ≤2 | 8 | **>2** | ≤1 | ≤1 | ≤1 | **≥32** | **>4** | **16** | ≤1 | |
| J53 | ≤2 | ≤4 | ≤2 | ≤1 | ≤1 | ≤1 | ≤8 | ≤1 | ≤2 | ≤4 | |
| J53/ICE*Pmi*Chn3725 | ≤2 | **>8** | **>2** | ≤1 | ≤1 | 4 | **>16** | ≤1 | **>32** | **>16** | |
| *E. coli* ATCC25922 | ≤2 | ≤1 | ≤20 | ≤0.25 | ≤0.25 | ≤1 | ≤4 | ≤0.25 | ≤1 | ≤1 | |

^a^ AMK, amikacin; GEN, gentamicin; SXT, trimethoprim/sulfamethoxazole; MEM, meropenem; IMP, imipenem; CAZ, ceftazidime; SAM, ampicillin/sulbactam; CIP, ciprofloxacin; CTX, cefotaxime; ATM, aztreonam. Resistant MIC′s are highlighted in bold.

**Table S8 MICs for FZP3105, its transformant and the recipient strain EC600.**

| **Strains** | **MIC (μg/ml) ^a^** | | | | | | | | | |
| --- | --- | --- | --- | --- | --- | --- | --- | --- | --- | --- |
|  | **AMK** | **GEN** | **SXT** | **MEM** | **IMP** | **CAZ** | **SAM** | **CIP** | **CTX** | **ATM** |
| FZP3105 | ≤2 | 8 | **≥320** | **16** | **8** | **32** | **≥32** | **≥4** | **≥64** | ≤1 |
| EC600 | ≤2 | ≤4 | ≤2 | ≤1 | ≤1 | ≤1 | ≤8 | ≤1 | ≤2 | ≤4 |
| EC600/pNDM_FZP3105 | **>32** | **>8** | **>2** | **8** | **4** | **>16** | **>16** | ≤1 | **>32** | ≤4 |
| *E. coli* ATCC25922 | ≤2 | ≤1 | ≤20 | ≤0.25 | ≤0.25 | ≤1 | ≤4 | ≤0.25 | ≤1 | ≤1 |

^a^ AMK, amikacin; GEN, gentamicin; SXT, trimethoprim/sulfamethoxazole; MEM, meropenem; IMP, imipenem; CAZ, ceftazidime; SAM, ampicillin/sulbactam; CIP, ciprofloxacin; CTX, cefotaxime; ATM, aztreonam. Resistant MIC′s are highlighted in bold.


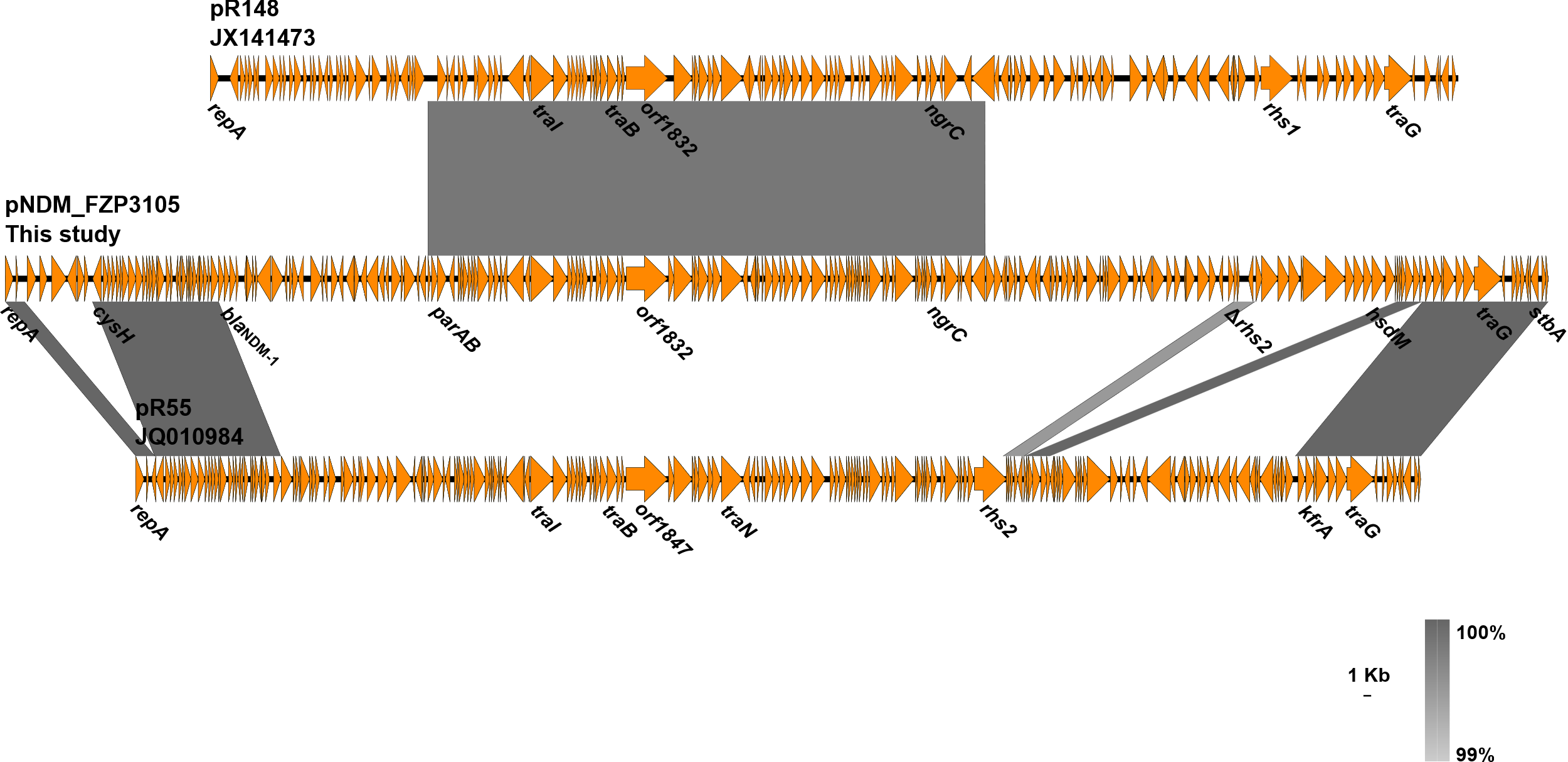


**Figure S1 Genetic structure of pNDM_FZP3105 and comparisons to related plasmids.** Arrows indicate deduced ORFs and their orientations. Gray shading denotes regions of shared homology ranging from 99% to 100%. Δ represents truncated genes.
